# Study of Flexural and Crack Propagation Behavior of Layered Fiber-Reinforced Cementitious Mortar Using the Digital Image Correlation (DIC) Technique

**DOI:** 10.3390/ma14164700

**Published:** 2021-08-20

**Authors:** Shaoqiang Meng, Jiaming Li, Zhihao Liu, Wenwei Wang, Yanfei Niu, Xiaowei Ouyang

**Affiliations:** 1 Research Center for Wind Engineering and Engineering Vibration, Guangzhou University, Guangzhou 510006, China; 2111916027@e.gzhu.edu.cn (S.M.); 2112016168@e.gzhu.edu.cn (J.L.); 2112016187@e.gzhu.edu.cn (Z.L.); 2112016225@e.gzhu.edu.cn (W.W.); 2 School of Civil Engineering, Guangzhou University, Guangzhou 510006, China

**Keywords:** layered concrete, FRCM, crack propagation behavior, DIC, interface

## Abstract

By optimizing the distribution of steel fibers in fiber-reinforced cementitious mortar (FRCM) through the layered structure, the role of fibers can be fully utilized, thus improving the flexural behavior. In this study, the flexural behavior of layered FRCM at different thicknesses (25 mm, 50 mm, 75 mm, 100 mm) of the steel fiber layer was investigated. The evolution of the crack propagation behavior was analyzed using the digital image correlation (DIC) technique. The results showed that the steel fiber layer thickness of 75 mm has the best flexural behavior. Moreover, the crack propagation path is more tortuous. The maximum value of crack opening displacement (COM) increases with the increase in fiber thickness. In addition, increasing the bottom layer thickness can increase the height of the tensile zone, but the interface inhibits the increase of the tensile zone.

## 1. Introduction

Fiber-reinforced cementitious mortar (FRCM) has attracted increasing attention due to its high strength, excellent flexural behavior, and crack control ability [1,2,3]. However, the high cost of raw materials for FRCM limits its widespread use in the engineering field [4,5]. To expand the application of FRCM, reducing its raw material cost has become an important research subject in this field. Using low-cost fillers and aggregates to prepare a FRCM matrix can reduce its raw material costs. The preparation of cementitious materials using waste fillers has been proven to be feasible [6,7,8,9]. Many scholars [10,11,12] have reported that the use of low-cost aggregates to prepare FRCM can reduce the cost without a significant loss of strength. Likewise, the high cost of steel fibers is a factor that cannot be ignored. Steel fibers account for 30–50% of the raw material cost of FRCM [13]. The cost of FRCM can be greatly reduced by increasing the efficiency of steel fiber reinforcement.

Under bending load, the region near the loading is mainly subjected to compressive stress, and the bottom bending region is mainly subjected to tensile stress. Steel fibers can significantly increase the tensile strength in the tensile region, but the contribution of steel fibers to the compressive strength is not significant compared to the tensile strength. The reinforcement efficiency of the steel fibers can be improved by optimizing the distribution of steel fibers in FRCM through a layered design. This design can reduce the cost of FRCM while improving flexural behavior [14].

Flexural behavior is a key indicator to evaluate the behavior of FRCM [15,16]. However, most studies have mainly focused on single-layer FRCM, while research on multilayer FRCM has rarely been reported. Lopez et al. [17] believe layered reinforced concrete has a similar damage pattern to ordinary reinforced concrete. Yue et al. [18] believe the flexural behavior of the designed double-layered beams is related to the thickness of the SHCC layer. Lai et al. [19] prepared a three-layer functionally graded cementitious composite (FGCC) and indicate that FGCC can improve flexural behavior and control the development of damage. Besides, the flexural behavior of FRCM is closely related to the crack propagation behavior. The crack propagation is influenced by fiber bridging and strain distribution in the fracture process zone [20,21,22]. To further evaluate the flexural behavior of layered FRCM, the crack propagation behavior needs to be investigated.

The usual methods to observe the crack propagation behavior are acoustic emission, swept surface electron microscopy, and cloud pattern method. Paul et al. [23] used acoustic emission and ARAMIS to accurately detect the fracture behavior of matrix and fibers in FRC. However, these methods require high testing environment and are difficult to perform full-field measurements in ordinary laboratories. The digital image correlation (DIC) technique has the advantages of full-field non-contact measurement and high accuracy, thus is widely used to observe crack propagation behavior [24,25,26]. For example, Ghafoori et al. [27] accurately quantified the crack propagation behavior of FRCM by the DIC technique. Therefore, the DIC technique is introduced to study the crack propagation behavior of FRCM.

In this study, the FRCM beams are designed as two layers of different thicknesses. Steel fiber-doped FRCM and FRCM matrix are the bottom layer and top layer, respectively. The flexural behavior is studied by the three-point bending test. Then, the crack propagation behavior is analyzed by the DIC technique. The crack propagation behavior of layered FRCM beams with different thicknesses is investigated. Finally, the research value of layered FRCM was evaluated in terms of its manufacturing process and mechanical properties.

## 2. Experimental Program

### 2.1. Materials and Mix Design

The mix design of the FRCM is listed in Table 1. P.II 52.5R Portland cement, quartz powder, and silica fume were used as cementitious materials. The chemical composition of cementitious materials is summarized in Table 2. Two particle sizes of quartz sand (0.212–0.425 mm and 0.425–0.710 mm) were used. The water-binder ratio (W/B) was 0.19. A superplasticizer was used to improve the workability of FRCM with a solids content of 33%. Steel fibers were incorporated to reinforce the flexural behavior of FRCM. The length of steel fiber is 13 mm and the diameter is 0.2 mm. The fiber doping is 2% (volume fraction). Its characteristic information is shown in Table 3.

The manufacturing process of FRCM is as follows: First, cement, quartz powder, and silica powder were dry mixed for 2 min. Then, the two-grain sizes of quartz sand were added to the dry mix, and low-speed stirred for 2 min to obtain a uniform mixture. Third, water and superplasticizer were mixed into a new liquid. Fourth, half of the liquid was poured into the mixture and low-speed stirred for 1 min, and the remaining liquid was poured into the mixture and high-speed stirred for 5 min. Once the mortar presented sufficient fluidity, the steel fibers were carefully dispersed into the fresh mortar. High-speed stirring was conducted for about 5 min until it appeared well distributed (based on visual appearance).

**Figure 1:**
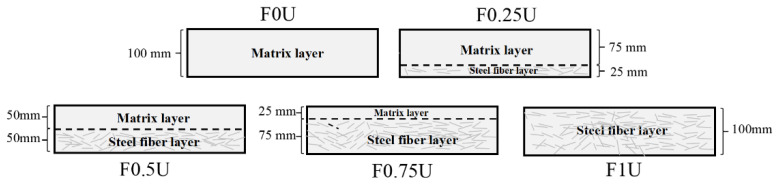
The specimen model.

The specimen was divided into two layers, the bottom layer was FRCM containing fibers and the top layer was FRCM matrix. Figure 1 presents the models of the five specimens considered, as suggested by Paulino et al. [28]. For instance, F0.25U is a double-layered FRCM, of which the bottom thickness is 25 mm and the top layer thickness is 75 mm. The FRCM containing fibers were poured into the bottom of the plastic mold from one end of the specimen to the other using a wide scoop. Then, the FRCM matrix was poured into the top of the plastic mold in the same way. Compared to extrusion, this casting process results in a stronger interfacial bond between the two layers, such that debonding does not occur under load. In addition, the interface creates micro-cracks under load, consuming more energy during the crack expansion process. Considering the effect of surface water evaporation on the bonding strength of the interface, the casting time interval was 10 min [29]. Specimens were covered with plastic sheeting to prevent the evaporation of surface water. The specimen was placed in a room with a temperature of 20 ± 5 °C for 24 h and cured in a water tank for 28 days after demolding.

### 2.2. Testing Methods

#### 2.2.1. Compressive Test

The compressive strength test was conducted on a universal testing machine (UTM) (Shisheng heng Machinery Manufacturing Co., LTD, Shanghai, China) with a maximum load capacity of 3000 KN. Loading direction is perpendicular to the interface. The size of the specimen used was 100 mm × 100 mm × 100 mm. The test temperature was 20 ± 5 °C. A loading rate of 0.8 MPa/s was used according to the ASTM standards C 39 [30]. The recorded value is the average of three specimens.

#### 2.2.2. Flexural Testing

**Figure 2:**
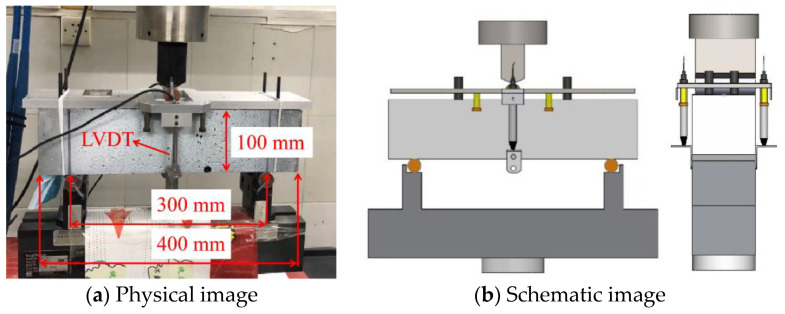
Three-point bending set-up.

A three-point bending test was conducted on a material testing system (MTS) using a servo-controlled electro-hydraulic machine (Shisheng heng Machinery Manufacturing Co., LTD, Shanghai, China). The loading setup is shown in Figure 2. A specimen with dimensions of 100 mm × 100 mm × 400 mm and a span of 300 mm was selected, as in the previous work [24,25]. Loading direction is perpendicular to the interface. The test temperature is 20 ± 5 °C. Two steel frames were placed at 1/2 of each side of the specimen, and a linear variable differential transformer (LVDT) was attached to each frame. The deflection will be accurately recorded through two LVTDs. The displacement control under a rate of 0.2 mm/min was used following the ASTM standards C1609. The roughness of the specimen surface and the bending of the specimen under load can have an impact on the test results. Therefore, a steel frame was installed on the specimen to ensure the accuracy of the test data. The bending equivalent stresses are expressed according to Equation (1):

(1) $$f=P\times \frac{L}{{\mathrm{bh}}^{2}}$$

where *L* is the span of the beam, b the height of the beam, and h the width of the beam.

#### 2.2.3. Digital Image Correlation

**Figure 3:**
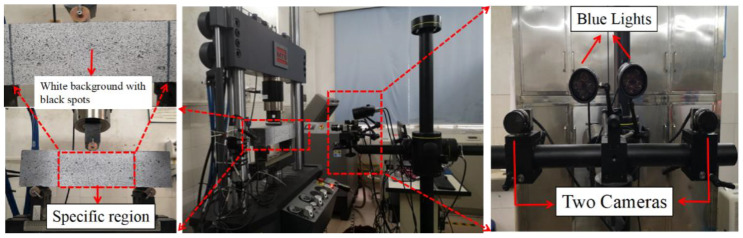
Test configuration of the DIC technique.

DIC is a method to calculate the displacement of points in a specific region [29]. As shown in Figure 3, two high-resolution cameras (Dantec LTD, Kässbohrerstr, Germany) with a focal length of 50 mm (1024 × 1024 pixels) were used to test the sample surface with scatter patterns. The lens shooting angle is 45°. To eliminate the influence of other light sources on the test results, two blue lights were used. The measured area was 100 mm × 200 mm. The loading direction is perpendicular to the interface of the specimen. The test temperature is 20 ± 5 °C. First, the scatter pattern before and after the deformation of a specific region during the flexural loading was acquired. Then, a grayscale analysis was performed in combination with a specific algorithm, which obtains the strain field of the region. The images were acquired in 1 s/frame. Detailed information about the DIC system can be found in the literature [30].

## 3. Results and Discussion

### 3.1. Compressive Strength

**Figure 4:**
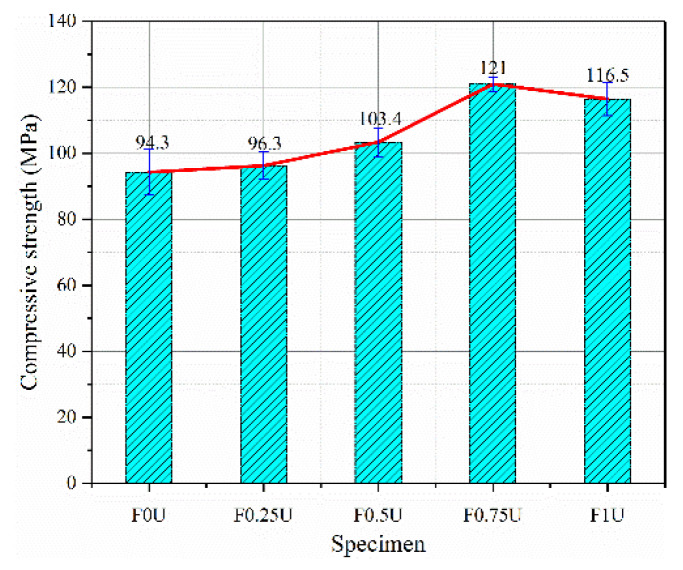
The compressive strength of various bottom layer thicknesses.

The compressive strength of layered FRCM with different thicknesses is shown in Figure 4. The recorded value is the average of three specimens. The compressive strength of the specimen increases with the increase in the thickness of the bottom layer until 75 mm. The compressive strength reaches the maximum at the thickness of 75 mm, which is 121.0 MPa. The compressive strength of F0U is 94.3 MPa, the compressive strengths of F0.25U, F0.5U, F0.75U, and F1U increased by 2.1%, 9.6%, 28.3%, and 23.5%, respectively.

### 3.2. Flexural Behavior

#### 3.2.1. Load-Deflection Curve

**Figure 5:**
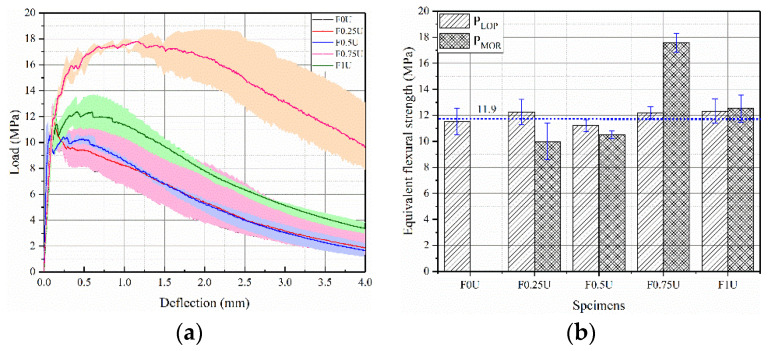
Load-deflection curves (**a**) and equivalent flexural strength (**b**) of specimens.

The load-deflection curves are shown in Figure 5a. Each curve is an average from three parallel samples. In the initial stage, the load increases linearly with the increase in deflection. When the deflection increases to a certain value, the first crack appears. During loading, the limit of proportionality (LOP) is defined as the initial crack strength, and the modulus of rupture (MOR) is defined as flexural strength [31,32]. Figure 5b shows the equivalent flexural strength of specimens. The value of P_LOP_ is about 11.9 MPa. The equivalent flexural strength (P_MOR_) of F0.25U, F0.5U, F0.75U, and F1U are 9.987 MPa, 10.508 MPa, 17.576 MPa, and 12.5217 MPa, respectively.

The initial crack strength of F0U and F0.25U is greater than that of the flexural strength (P_LOP_ > P_MOR_), which is a deflection softening behavior. The flexural strength at the initial crack strength of F0.75U, F1U is greater than that of the flexural strength (P_LOP_ < P_MOR_), which is a deflection hardening behavior. In other words, the bottom thickness is less than 50 mm for deflection softening behavior and more than 50 mm for deflection hardening behavior. This is due to the thin bottom fiber layer (<50 mm), which results in a weak bridging effect provided by the fibers. The flexural strength is mainly provided by the bridging effect of the aggregates. As the thickness of the bottom layer increases (>50 mm), the bridging effect of the fibers can effectively inhibit crack propagation, leading to an increase in flexural strength.

#### 3.2.2. Deflection Capacity and Toughness

**Figure 6:**
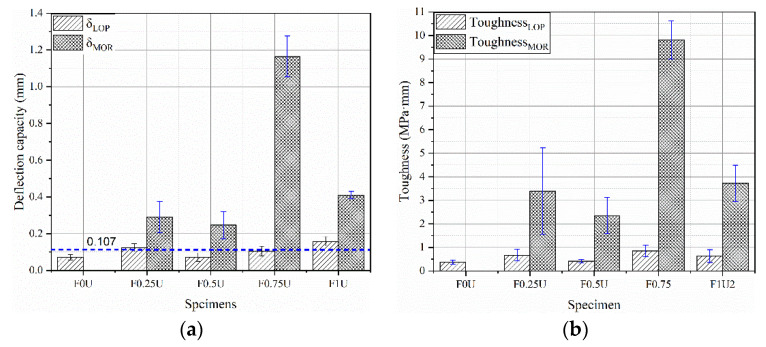
Deflection capacity (**a**) and toughness (**b**) of specimens.

The deflection capacity of layered FRCM with different thicknesses is shown in Figure 6a. The value of δLOP is about 0.107 mm. The toughness can be calculated from the area enclosed by the deflection curve. The toughness of layered FRCM with different thicknesses is shown as Figure 6b. The average value of Toughness_LOP_ is 0.641 MPa·mm. This is due to the flexural behavior before cracking being provided by the FRCM matrix, independent of the fiber [27,33,34]. The toughness of F0U, F0.25U, F0.5U, F0.75U, and F1U are 0.366 MPa·mm, 3.395 MPa·mm, 2.349 MPa·mm, 9.809 MPa·mm, and 3.718 MPa·mm, respectively.

**Figure 7:**
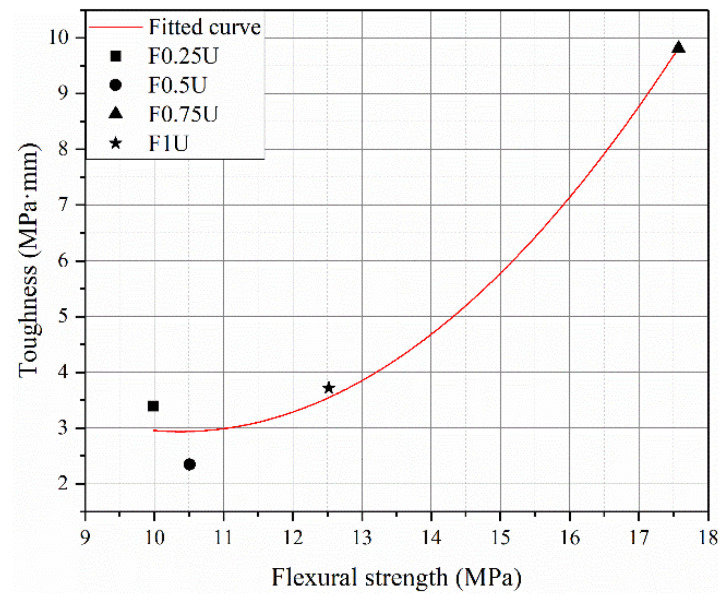
The relationship between flexural strength and toughness.

By studying the relationship between flexural strength and toughness (Figure 7), it was found that a quadratic polynomial can accurately represent this relationship, as shown in Equation (2). The R^2^ is 98.3%.

(2) $$y=-2.76x^{2}+0.133x+17.26$$

where *x* is the flexural strength and y is the toughness.

### 3.3. Analysis of Crack Propagation Behavior

#### 3.3.1. Quantification of the Strain Fields

DIC analysis was performed for layered FRCM under different loading stages. Since F0U is a brittle failure, it is difficult for the DIC technique to capture its crack propagation behavior. Therefore, the detailed discussion of this study is limited to the strain fields in the specimens reinforced by fiber layers.

**Figure 8:**
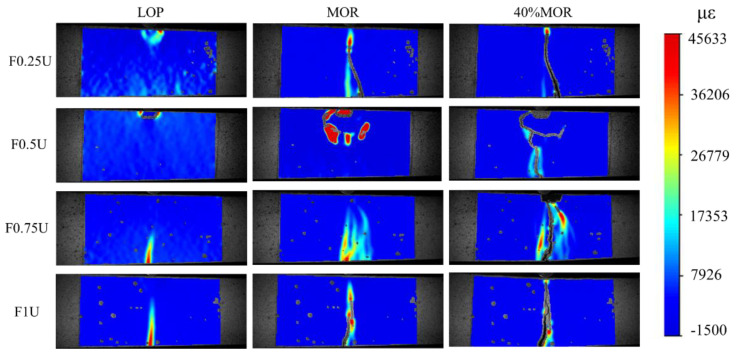
Crack patterns and strain distribution of specimens under different loads.

Crack patterns and strain distribution of layered FRCM under different loading stages is shown in Figure 8. At the LOP point, the first cracks of F0.25U and F0.5U appear at the top, and these cracks are mainly concentrated near the loading point. However, the first cracks of F0.75U and F1U appear at the bottom. It was also found that F1U has a longer strain region than F075U.

At the MOR point, the main crack in F0.25U changes to develop from the bottom. The crack at the top of F0.5U continues to develop down to about 50 mm. The crack in F0.75U continues to develop upward to about 75 mm. The length of the cracks in F1U exceeds 75 mm.

At the 40% MOR point (post-flexural strength stage), the crack width of F0.25U increases while the cracks develop further upward. Cracks developed from the bottom appear in F0.5U, and these cracks converge with the cracks generated from the top at 50 mm. Cracks in F0.75U continue to develop upward, but the increase in length is not significant. Meanwhile, the top concrete reaches the ultimate compressive strain and cracks appear. Cracks in F1U develop upward in a jagged pattern.

In conclusion, when the thickness of the bottom steel fiber layer is less than 50 mm, the first cracks appear at the top layer. This is due to the low compressive strength caused by the thicker top layer, and thus the top layer undergoes compression damage. Long cracks developed from the bottom early in F0.25U due to the small fiber layer at the bottom. Crack development at 50 mm laterally in F0.5U may be related to the interface between the upper and lower layers. When the thickness of the bottom steel fiber layer is more than 50 mm, the strain distribution is wider and multiple crack branches are observed. F0.75U has the more complex strain and crack paths compared to F1U. The cracks in F0.75U finally extended to about 75 mm. This may be due to the interface between the bottom and top layers preventing the transfer of strain. Of course, this remains to be further analyzed.

#### 3.3.2. Quantification of COD Evolution

**Figure 9:**
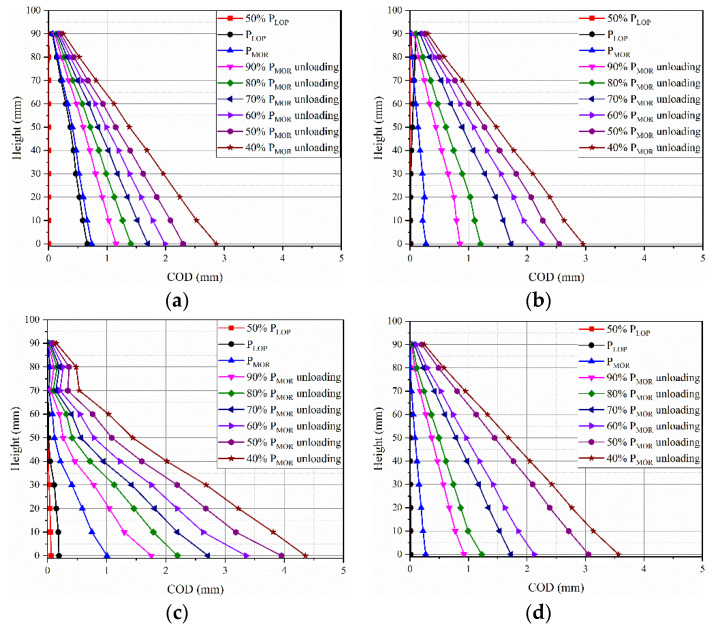
COD evolution in fracture tests with perpendicular orientation in F0.25U (**a**), F0.5U (**b**), F0.75U (**c**), and F1U (**d**).

To evaluate the toughness of layered FRCM with different thicknesses, the crack opening displacement (COD) evolution of the specimens is analyzed. The COD are quantified by the displacement field under different loading stages. Figure 9 shows the evolution of COD under different loading stages. It can be seen that the height is linear as a function of COD in F0.25U (Figure 9a), F0.5U (Figure 9b), and F1U (Figure 9d). The COD is not linear as a function of height in F0.75U (Figure 9c). This indicates that F0.75U consumes more energy under bending load.

The maximum value of COD for F0.25, F0.5U, F0.75U, and F1U is 2.87 mm, 2.95 mm, 4.36 mm, and 3.56 mm, respectively. It is further shown that increasing the thickness of the steel fiber layer can effectively imprint the propagation behavior of FRCM. This result is matched with the results of LVDT.

#### 3.3.3. Quantification of Strain Distribution

**Figure 10:**
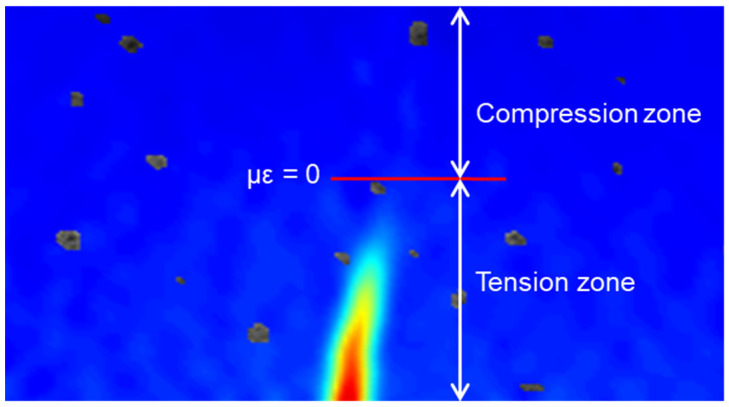
Schematic diagram of the compression and tension zones.

**Figure 11:**
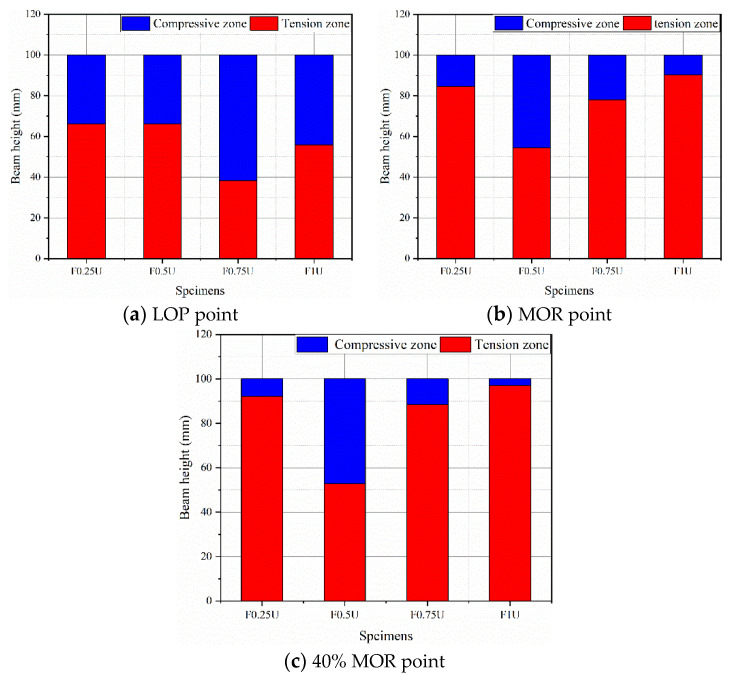
Compressive and tensile zones at LOP point (**a**), MOR point (**b**), and 40%MOR point (**c**).

To analyze the role of the interface between the bottom and top layers of the layered FRCM under loading stages, the distribution of the longitudinal strain zones will be discussed in this section. The positive value of strain indicates the tensile zone, and the negative value indicates the compressive zone, as shown in Figure 10. Figure 11 presents the distribution of the longitudinal strain zones of layered FRCM with different thicknesses under various loads. The distribution of the longitudinal strain zones of layered FRCM at the LOP point is shown in Figure 11a. The tensile zones of F0.25U and F0.5U are similar at 66.2 mm. The tensile zone of F0.75U is 38.2 mm, which is the smallest. The height of the tensile zone of F1U is 55.9 mm, which is greater than F0.75U.

Figure 11b demonstrates the distribution of the longitudinal strain zones of layered FRCM at the MOR point. The tensile zone of F0.25U is 84.5 mm. Although the first cracks in F0.25U started from the top layer, the cracks quickly developed from the bottom due to the small thickness of the bottom layer. Subsequently, the cracks developed rapidly upward, leading to the high tensile zone. The crack continues downward from the top layer in F0.5U. The strain transfer reaches the interface (about 50 mm) and then the transfer is restricted. The F0.75U crack develops from the bottom and the bottom fibers transfer the strain upward to the interface (about 75 mm). It is indicated that the influence of the interface prevents strain from further development. Strain in the F1U is rapidly transferred upward from the bottom, resulting in a high stretch zone (90.2 mm).

The distribution of the longitudinal strain zones of layered FRCM at the 40% MOR point is shown in Figure 11c. At this time, the cracks in F0.25U, F0.75U, and F1U develop further. The tensile zone further increases to 90.2 mm, 88.6 mm, and 97.1 mm, respectively. The continuous increase in load leads to cracking of the top layer of concrete by reaching ultimate compressive strain, which converges with the cracks generated by tensile strain at the interface (about 50 mm). It is worth noting that the height of the tensile zone of F0.5U and F0.75U is approximately the height of their interface.

In short, for bottom fiber layer thickness less than 50 mm, the flexural behavior is mainly contributed by the bridging effect of the aggregate, resulting in the high height of the tensile zone at the LOP point. After reaching the MOR point, the height of the tensile zone of F0.25U continues to increase. However, F0.5U maintains the height of the tensile zone at 50 mm due to the presence of the interface that hinders the transfer of strain. When the thickness of the bottom fiber layer is greater than 50 mm, the bending load transitions to be mainly carried by the fibers, causing the tensile zone to be smaller. Similarly, the tensile zone of F0.75U is finally kept at about 75 mm after reaching the MOR point. Since F1U has no interface, the transfer of strain is faster, leading to a higher tensile zone than F0.75U.

In other words, the strain distribution of layered FRCM is mainly controlled by two factors: the thickness of the bottom fiber layer and the interface between the two layers. When the bottom fiber layer is 25 mm and 100 mm, the thickness of the bottom fiber layer is the dominant factor affecting the strain distribution. When the bottom fiber layer is 50 mm and 75 mm, the strain distribution is controlled by the combined effect of the bottom fiber layer thickness and the interface.

## 4. Conclusions

In this study, FRCM beams are designed in layers with different thicknesses. The flexural behavior was investigated by a three-point bending test. Then, the crack propagation behavior was analyzed using the DIC technique. The effect of bottom layer thickness on the crack propagation behavior was discussed in terms of crack and strain paths, COD, and distribution of local strain fields. Based on the obtained results, we may conclude the following:

(1) Increasing the thickness of the steel fiber layer can increase the equivalent flexural strength and toughness until 75 mm. The bottom steel fiber layer is less than 50 mm for flexural softening behavior and more than 50 mm for flexural hardening behavior.

(2) The increase of the thickness of the steel fiber layer makes the crack development path more curved. The phenomenon of multiple crack branches appears when the thickness of the bottom layer is 75 mm. The expansion of cracks into the interface will be limited by the interface.

(3) The height of the tensile zone is related to the thickness of the bottom fiber layer and the interface. For F0.25U and F1U, the thickness of the bottom fiber layer is the dominant factor affecting the strain distribution. For F0.5U and F0.75U, the strain distribution is controlled by the combined effect of the bottom fiber layer thickness and the interface.

In this study, a detailed analysis of the crack expansion behavior of layered FRCM was conducted to confirm the advantages of layered FRCM. The design of layered FRCM can provide a new way to study reducing the cost of FRCM and improving its flexural behavior. However, the sample fabrication method in this study is labor-intensive and difficult to apply in practical engineering. Therefore, The extrusion process may be a better choice for engineering applications. The strain field at the interface using extruded layered FRCM will be further studied.

## Figures and Tables

**Table 1:** Mix proportions of FRCM (kg/m^3^).

Materials	Cementitious Materials			QS		W	SP	Fiber
	CEM	QP	SF	0.212–0.405 mm	0.425–0.710 mm			
Quantity	998.52	302.15	129.75	250.58	751.75	257.52	18.47	2%

[Note]: CEM = portland cement, SF = silica fume, QP = quartz powder, QS = quartz sand, W = water, SP = superplasticizer.

**Table 2:** The chemical compositions of cementitious materials (%).

Compound	CaO	SiO_2_	Al_2_O_3_	Fe_2_O_3_	SO_3_	Other
CEM	62.22	21.17	6.43	3.67	3.04	3.105
SF	0.431	96.94	6.43	−	0.124	1.567
QP	0.0117	97.8	1.67	0.0271	0.0044	0.3339

**Table 3:** Steel fiber properties.

Diameter d_f_ (mm)	Length l_f_ (mm)	Aspect Ratio (l_f_/d_f_)	Density (g/cm^3^)	Tensile Strength (MPa)	Elastic Modulus (GPa)
0.2	13.0	65.0	7.8	2500	200

## Data Availability

The associated data in this study are available from the corresponding author.
